# Evaluation of Biosafety, Antiobesity, and Endothelial Cells Proliferation Potential of Basil Seed Extract Loaded Organic Solid Lipid Nanoparticle

**DOI:** 10.3389/fphar.2021.722258

**Published:** 2021-10-04

**Authors:** Pandurangan Subash-Babu, Nada Al-Saran, Ghedeir M. Alshammari, Laila Naif Al-Harbi, Maha Hussain Alhussain, Ghalia Shamlan, Sahar Abdulaziz AlSedairy, Ali Abdullah Alshatwi

**Affiliations:** Department of Food Science and Nutrition, College of Food and Agricultural Sciences, King Saud University, Riyadh, Saudi Arabia

**Keywords:** basil seed, SLNPs, angiogenesis, mitochondria, vascular disease, lipolysis

## Abstract

The present study aimed to synthesize solid lipid nanoparticles to enhance liposome-assisted intracellular uptake of basil seed active components in adipocytes and vascular smooth muscle cells to attain increased bioavailability. To obtain solid lipid nanoparticle (SLNp), the water phase containing basil seed extract (BSE) was encapsulated with lipid matrix containing chia seed phospholipids using homogenization and cold ultra-sonication method. The physicochemical characterization of BSE loaded solid lipid nanoparticles (BSE-SLNp) has been analyzed using Zetasizer, FT-IR, and TEM. The BSE-SLNp showed an average diameter of 20–110 nm on the day of preparation and it remains the same after 60 days of storage. The cytotoxicity assay confirmed that the BSE-SLNp did not produce toxicity in hMSCs, preadipocytes, or human umbilical vein endothelial cells (HUVECs) until the tested higher dose up to 64 μg/ml. During effective dose determination, 4 μg/ml of BSE-SLNp confirmed non-toxic and enhanced metabolic function in hMSCs, preadipocytes, and HUVECs. Biosafety assay confirmed normal nuclear morphology in PI staining and high mitochondrial membrane potential in JC-1 assay within 48 h in hMSCs. The maturing adipocyte treated with 4 μg/ml of BSE-SLNp significantly increased the mitochondrial efficiency and fatty acid beta-oxidation (PPARγC1α, UCP-1, and PRDM-16) related gene expression levels. Oxidative stress induced HUVECs treated with 4 μg/ml of BSE-SLNp potentially enhanced antioxidant capacity, cell growth, and microtubule development within 48 h H_2_O_2_ induced oxidative stressed HUVECs have shown 39.8% viable cells, but treatment with BSE-SLNp has shown 99% of viable cells within 48 h confirmed by Annexin-V assay. In addition, mitochondrial membrane potential (Δψ_m_) increased to 89.4% confirmed by JC-1 assay. The observed DNA integrity, cell viability was confirmed by increased antioxidant and tumor suppressor-related gene expression levels. VEGF expression has been significantly increased and pro-inflammation-related mRNA levels were decreased in BSE-SLNp treated cells. In conclusion, enhanced adipocyte fatty acid oxidation is directly associated with decreased adipocytokine secretion which arrests obesity-associated comorbidities. In addition, suppressing vascular cell oxidative stress and metabolic inflammation supports vascular cell proliferation and arrests ageing-related vascular diseases.

## Introduction

Pathogenesis of age-related chronic complications is majorly dependent on the functional and structural modifications of the vascular system on metabolic stress. Aging progress encounters impairment of angiogenesis in endothelial cells, apoptosis, and decline in capillary regression, which all contribute to declining tissue perfusion (Gardner et al., 2016). Altered endothelial cells develop proinflammatory cytokines such as eNOS, ICAM, and VCAM were associated with the development of age-associated chronic inflammatory diseases such as atherosclerosis, osteoarthritis, metabolic diseases, and gastrointestinal tract complications (Al-Khazraji et al., 2018). Aging-induced deregulation of endothelial barrier breaching and transport function initiates the leakage of microbial metabolites into circulation and contributes to chronic, irreversible, low-grade inflammation and distant organ damage (Spadoni et al., 2015).

The aging process reduces mitochondrial oxidative efficiency, reduces biogenesis, mutation in mtDNA, and alters mitochondrial membrane potential (dynamics) or defective mitophagy (Sun et al., 2016). Reduced mitochondrial efficiency decreases fatty acid beta-oxidation and energy production, promoting TG accumulation which ends with obesity. A high-fat diet substantially arrests AMPK phosphorylation in white adipose tissue, heart, and liver; it is directly associated with the progression of systemic insulin resistance, hyperlipidemia, and obesity (Lindholm et al., 2013). Subcutaneous or visceral obesity have been associated with a proinflammatory response, which can activate the negative consequences in stem cells (Onate et al., 2012). Active stem cells of subcutaneous adipose tissue derived from obese patients were found with a reduced proliferation ability, viability, change in telomerase activity, and reduced differentiation potential or loss of proangiogenic capacity (Perez et al., 2015). Chronic obesity impairs gene expression patterns related to cellular proliferation, differentiation, angiogenic potential, and multipotent capacity (Onate et al., 2013). Pharmacological agents or therapeutically functionalized nanoparticles target adipocyte mitochondrial efficiency and vascular cell proliferation attracts the aged obese population majorly.

Basil seed (*Ocimum basilicum* L.) has been used in traditional medicine and consumed as flavor and spices in the food industry worldwide. Basil seed leaf essential oil has antioxidant (Abd El-Ghffar et al., 2018) and anti-inflammatory potential (Takeuchi et al., 2020). Noor et al. (2019) have reported that 200 μg/ml of basil seed extract show antiobesity and anti-inflammatory potential. Akbarian et al. (2016) have found that the biological availability of orally supplemented basil seeds is very tracing in human subjects, which resulted in reduced biological efficiency. Many of the activity-established phyto principles have been rigorously restricted in their development because of their low solubility, diffusion, and bioavailability (Badawi et al., 2020). Identification and oral supplementation of bioactive compounds for mitochondrial stimulation, such as lipolytic potential with the functional stimulation of β-adrenergic receptor followed by the activation of AMPK and CREBp-1, became very challenging because its low intracellular uptake ends with low biological availability and loss of efficiency. The bioactive compound across the cellular lipid bilayer without covalent modification or conformational change may provide maximum efficiency and deserve their application in obesity treatment. In the modern approach, the applications of SLNp as a carrier in facilitating the effective diffusion through the cell membrane increased the bioavailability of active ingredients such as silymarin, berberine, triptolide, metformin, and pomegranate extract for antiobesity and anti-inflammatory treatment, which is most successful (Xue et al., 2015; Mohseni et al., 2019; Badawi et al., 2020). We have selected chia seed phospholipid as the encapsulating material because of its recommended ratio of omega 3 and omega 6 with lipolytic potential (Pandurangan et al., 2020). Chia seed mucilage has been considered an alternative to synthetic polymers because it was identified and achieved as an encapsulating material with the highest encapsulation efficiency and loading capacity (de Campo et al., 2017).

**Image 1:**
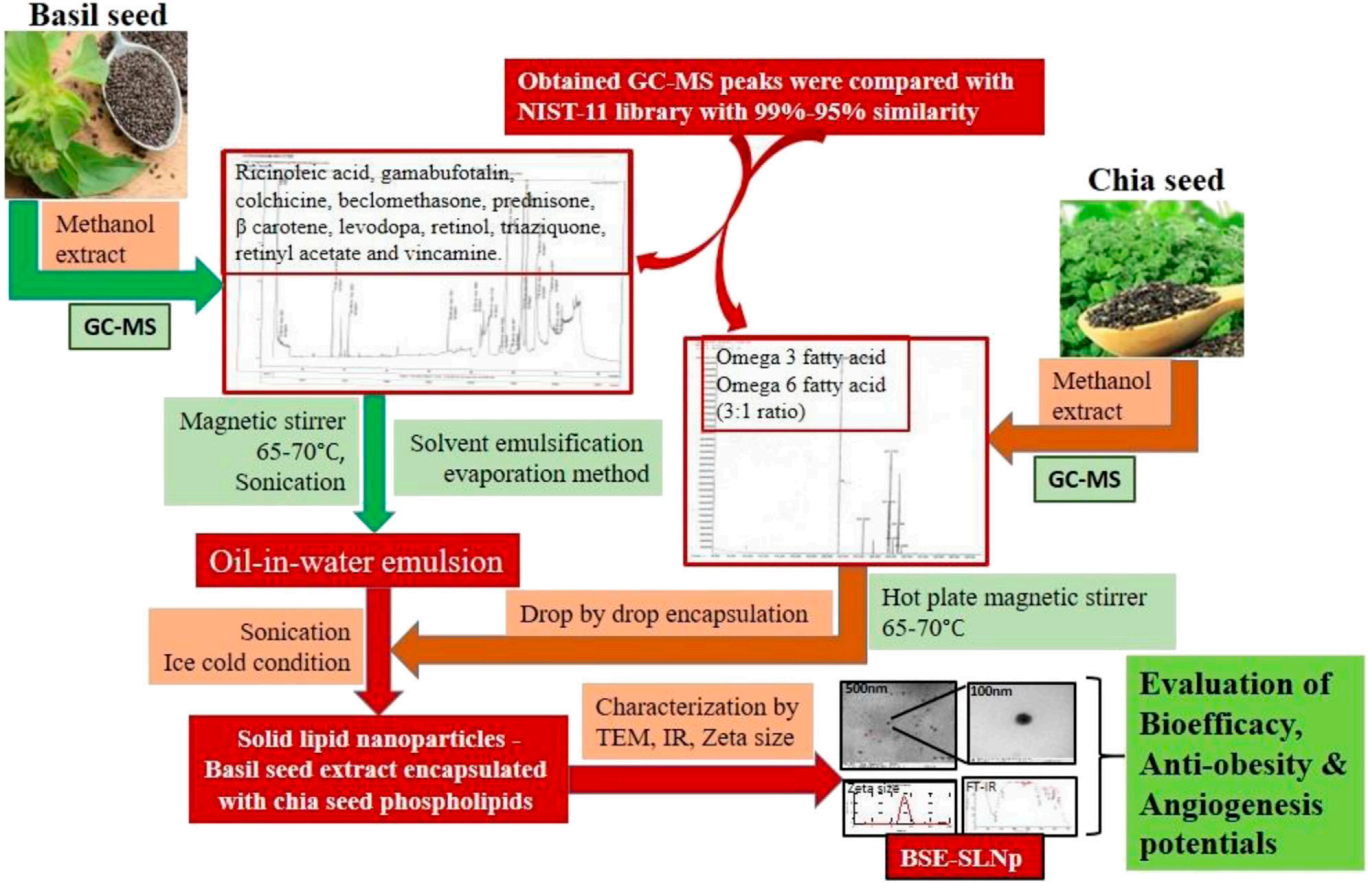
Diagrammatic illustration for the process of BSE-SLNp preparation and characterization.

Image 1 | Diagrammatic illustration for the process of BSE-SLNp preparation and characterization.

SLNp with submicron particle size (30–200 nm) has been recognized as a potential carrier system as liposomes or emulsions (Muller et al., 1995). SLNp is most suitable as carrier material without showing any adverse effect and protecting the loading materials from chemical digestion or physiological damage compared to other drug carriers (Garg et al., 2012). We aimed to encapsulate the BSE water phase with a lipid matrix containing chia seed phospholipids in the present study. We studied its toxicity in human mesenchymal stem cells (hMSCs), lipolytic effect in maturing adipocytes, angiogenesis, and protective effect in oxidative stressed HUVECs.

## Materials and Methods

### Cell Culture Materials and Chemicals

hMSCs and HUVECs were obtained from American type culture collection (ATCC, Manassas, VA). DMEM (Dulbecco’s modified Eagle medium), EDTA (ethylenediaminetetraacetic acid), and trypsin were purchased from Gibco (Paisley, United Kingdom). FBS (fetal bovine serum) and penicillin-streptomycin were obtained from Hyclone Laboratories, U.S. MTT [3-(4,5-dimethylthiazol-2-yl)-2,5-diphenyltetrazolium bromide], propidium iodide, JC-1 stain, Oil Red O, Nile red, and all other chemicals related to molecular biology experiments were purchased from Sigma-Aldrich (St. Louis, MO).

Adipocyte differentiation factors, 3-isobutyl-1-methyl-xanthine (IBMX), rosiglitazone, dexamethasone (DEX), and insulin were purchased from Sigma (St. Louis, MO). The cell to cDNA synthesis kits and SYBR Green PCR master mix were obtained from Qiagen (Hilden, Germany). ELISA kits for proinflammatory cytokine were purchased from Qiagen (Hilden, Germany).

### Plant Material, Extraction of Basil Seed Active Principles and Chia Seed Phospholipids

Basil seed (*Ocimum basilicum* L.) and chia seed ( *Salvia hispanica* *.* L.) were purchased from the local market, Riyadh, Saudi Arabia. According to the national and international regulations, the plant species were identified and utilized in the present study. The speciess were identified and authenticated by a taxonomist (Dr. V. Duraipandiyan, Herbarium, Department of Botany and Microbiology, College of Science, King Saud University, Riyadh). The voucher specimen, such as KSU-OB-04 for basil seed (*Ocimum basilicum* L.) and KSU-SH-07 for chia seed ( *Salvia hispanica* *.* L.), were deposited and preserved in the Public Herbarium (College of Science, King Saud University, Riyadh).

Basil seed and chia seed were sieved, milled, and ground using a commercial blender. There was 400 gm of basil seed powder soaked in 1.2 L of methanol for 72 h with frequent shaking using a mechanical shaker. After 72 h, the components extracted in methanol were filtered and reconstituted at reduced pressure at 40°C using a rotary evaporator. Condensed BSE was filtered and stored at −20°C until further use. Separately, 200 gm of coarsely powdered chia seed was soaked in 600 ml of ethyl acetate for 72 h with frequent shaking using a mechanical shaker. The chia seed extract (CSE) reconstitution process has been carried out the same as the basil seed progress.

### Chemical Analysis Using GC-MS

The phytochemical content of BSE and CSE were analyzed using an Agilent 7890A (Agilent Technologies, U.S.) gas chromatography (GC) coupled with a 5,975 inert mass-spectrometer (MSD). The system was equipped with a DB-5MS GC column (30 m length, 0.25 mm inner diameter, and 0.25 µm film thickness), a Triple-Axis detector (MSD), and a 7,693 automated liquid sampler. One milliliter of particle-free diluted seed extracts was filtered through a 2-µm membrane filter and it was injected into the injector with a split ratio of 30:1. The initial injection temperature was set as 280°C and the column temperature was maintained as 300°C. Helium was used as the carrier gas with a flow rate of 1 ml/min. The electron ionization energy was 70 eV. All data were collected after the full-scan mass spectra within the scan range 40–550 AMU. The percentage composition of the BSE or CSE constituents were expressed as a percentage by peak area.

### Basil Seed Extract Loaded Solid Lipid Nanoparticle Preparation

SLNp have been fabricated by the solvent emulsification evaporation method, such as BSE-loaded with chia seed phospholipid (containing 3:1 ratio of omega 3:omega 6), phosphatidylethanolamine, palmitic acid, and stearic acid according to the method of Xue et al. (2015), with minor modifications. Briefly, BSE (5, 10, and 15 mg, respectively), solvent-free CSE-phospholipids (30 mg), phosphatidylethanolamine (0.25% w/w), palmitic acid (0.25% w/w), and stearic acid (0.25 mg) were dissolved in 5 ml of chloroform and methanol (2:1) and kept in a magnetic stirrer at 65°C for complete dissolving. The warm organic lipid phase has been emulsified with the aqueous phase containing Tween 80 (30 mg dissolved in 15 ml of water). The whole process was carried out at 70°C (above the melting point of the lipid) using a hot plate magnetic stirrer with constant stirring for 30 min. The coarse oil-in-water dispersion was sonicated at a frequency of 0.5 cycles and 60% amplitude using a probe-type Ultrasonicator (Sonics, U.S.) in an ice bath. Approximately 20 ml of the formulation contained 0.05% of the drug, 50 mg of the lipid phase and 15 ml of the aqueous phase. The obtained dispersion was collected and stored in a brown glass container and stored at 2–4°C in the freezer until further use.

### Characterization of Prepared BSE-SLNp

The total drug content in BSE-SLNp was estimated by disrupting 1 ml of BSE-SLNp dispersion in a freshly prepared and 3-months stored sample using a spectrophotometer at 420 nm. The chemical interaction with other excipients was recorded by FT-IR (Agilent, U.S.). The particle size was determined as the z-average diameter using the dynamic light scattering (DLS) technique using a Zetasizer (NANO-Zs90). To analyze the morphology, 5 μl of BSE-SLNp was loaded on the surface of a 300-mesh carbon-coated copper grid. There was 2% uranyl acetate (w/v) used as negative staining for the sample. Then, shape, size, and morphology were analyzed from the stained grids using high-resolution transmission electron microscopy (HR-TEM, JEOL, Japan).

### HUVECs, hMSCs Culture, and Adipocyte Differentiation

HUVECs and hMSCs were cultured in DMEM containing 10% fetal bovine serum and 100 U/mL penicillin-streptomycin at 37°C in a humidified 5% CO_2_ incubator. To induce adipocyte differentiation, hMSCs were seeded (2 × 10^4^ cells/well) in a 24-well plate and allowed to reach 80% confluence with the growth conditions. After 48 h, hMSCs (day 0) were replaced with adipocyte differentiation media containing DMEM with 10% FBS, 0.5 mM IBMX, 1 µM dexamethasone, and 10 μg/ml of insulin. On Day 3, cells were replaced with adipogenesis maintenance medium (DMEM with 10 μg/ml insulin) for 2 days (Pandurangan et al., 2020). Subsequently, cells growing in a maintenance medium were treated with drug preparations, according to the study. Experimental cells were replaced with fresh media once in 3 days until the experimental period. Cells maintained in the maintenance medium alone were used as a negative control.

### Cytotoxicity Analysis

HMSCs, preadipocytes, and HUVECs were maintained in log phase at 37°C and 5% CO_2_ and seeded at a concentration of 1 × 10^4^ cells/well in a 96-well plate. The cells were then incubated overnight to allow the cells to re-adhere to the plates. After discarding the growth medium, the freshly prepared BSE, CSE, and freshly sonicated BSE-SLNp (1, 2, 4, 8, 16, 32, and 64 µg/ml) were reconstituted in the desired medium and treated to respective well and incubated for 48 h; untreated cells were used as controls. After incubation, cells were gently washed with PBS, then 20 µl/well of MTT (5 mg/ml) [3-(4, 5-dimethylthiazol-2-yl)-2, 5-diphenyltetrazolium bromide] were added and incubated for 4 h at 37°C in a CO_2_ incubator. At the end of incubation, the purple formazan crystals were dissolved in 100 µl of 100% DMSO. The amount of formazan formed was determined by measuring the absorbance at 570 nm using a microplate reader (Thermo Scientific). Percentage of cell growth inhibition was calculated according to the mean values of (absorbance of the sample/absorbance of the control) × 100.

### Effective Dose Determination

HMSCs, preadipocytes, and HUVECs were maintained in log phase at 37°C and 5% CO_2_ and seeded at a concentration of 1 × 10^4^ cells/well in a 96-well plate and 5 × 10^4^ cells/well in a 24-well plate. Adherent cells were subsequently treated with 2, 4, and 8 μg/ml doses of freshly prepared BSE, CSE, and freshly sonicated BSE-SLNp reconstituted in the desired medium (dose selected based on cytotoxicity analysis). The working dilutions were prepared from the NP stock dispersion and added to 200 μl of cell suspension in a 96-well plate or 500 μl of cell suspension in a 24-well plate. After 48 h, the effective dose effect of BSE, CSE, and BSE-SLNp were determined based on a metabolically active cell’s morphological behavior with mitochondrial membrane potential (JC-1 assay).

### Experimental Design

To determine the biosafety of BSE-SLNp, hMSCs were treated with a 4-µg/ml concentration of BSE, CSE, and BS-SLNp for 48 h (4 µg/ml dose has been selected based on effective dose determination assay). Then, control and experimental cells were analyzed for cell and nuclear morphology, JC-1, and gene expression analysis. To determine the antiobesity potential in maturing adipocytes, preadipocytes were treated with increasing concentrations of BSE, CSE, and BSE-SLNp for 14 days, the regulatory effect and alterations in adipocyte’s fatty acid metabolism were analyzed. Initially (on Day 0), vehicle control, 4 μg/ml of BSE, CSE, and BSE-SLNp were treated to preadipocytes. On Day 3, BSE, CSE, and BSE-SLNp treated preadipocytes were replaced with a maintenance medium containing 4 μg/ml of BSE, CSE, and BSE-SLNp and maintained until Day 6. However, vehicle control was replaced with a maintenance medium without drugs. From Day 7 to Day 14, the media was replaced with maintenance medium once in 3 days.

To determine angiogenesis and apoptotic potential in HUVEC, 4 µg/ml concentration of BSE, CSE, and BSE-SLNp was treated to H_2_O_2_ induced oxidative stressed HUVEC for 48 h. Then, the untreated and experimental cells were processed for cell and nuclear morphology, mitochondrial membrane potential has been analyzed in flow cytometry using BD^TMNile red^ MitoScreen (JC-1) Kit, Annexin-v/apoptosis, cell cycle analysis, gene expression level, and ELISA-based protein quantification have been carried out.

At the end of the experimental cell’s condition media (containing BSE, CSE, and BSE-SLNp treated adipocyte secreted and cellular proteins) have been collected for protein quantification and the adherent cells were used for staining or cDNA synthesis for gene expression analysis.

### Cell and Nuclear Morphology

BSE, CSE, and BSE-SLNp treated cells were processed to determine the characteristic apoptotic and necrotic morphological changes using light microscopy or florescent microscopy [propidium iodide (PI) and acridine orange (AO)] described by Leite et al. (1999).

### Light (Oil Red O) and Fluorescent (Nile Red) Microscopy Cell Image Analysis

According to our previous study, Oil Red O and Nile red staining analysis were carried out (Pandurangan et al., 2020). Briefly, 500 mg of Oil Red O in 100 ml of 100% isopropanol were prepared as the stock solution and the working solution was prepared with a 3:2 ratio of stock with 60% isopropanol. We added 200 μl of working Oil Red O solution to 4% formaldehyde fixed vehicle control, BSE, CSE, and BSE-SLNp treated (4 μg/ml) maturing adipocytes. After 1 h of incubation at room temperature, free oil red’ Oil Red O was gently removed by PBS washing and immediately the images were analyzed using an inverted light microscope.

Fluorescent Nile red staining was performed to determine the characteristic features of hypertrophic adipocytes after treatment. Briefly, 4% formaldehyde fixed vehicle control, BSE, CSE, and BS-SLNp treated experimental cells were stained with Nile red fluorescence (5 mg in 1 ml of 100% acetone) for 30 min at 37°C. After incubation, the accumulation of fluorescence was captured immediately using an inverted fluorescence microscope.

### Mitochondrial Membrane Potential (Δψ_m_) (JC-1 Staining) Assay

Mitochondrial membrane potential (Δψ_m_) was determined using JC-1 dye in vehicle control, BSE, CSE, and BSE-SLNp treated adipocytes. In addition, mitochondrial membrane potential was measured in flow cytometry using BDTM MitoScreen (JC-1) Kit. Briefly, JC-1 staining solution (mixed with an equal volume of culture medium) was added to experimental cells and incubated for 20 min in the dark at 37°C. After incubation, the unbound JC-1 dye has been gently removed by washing with 200 μl of JC-1 staining wash buffer at 4°C and this process is repeated two times, then the morphology was observed under fluorescence microscopy. Then, the fluorescence was observed using fluorescence microscope and images were captured.

### Annexin V/Apoptosis Analysis Using Flow Cytometry

Annexin V/propidium iodide (PI) (Sigma Chemicals, U.S.) assay was carried out to quantify viable, apoptotic (early and late), and necrotic cells using flow cytometry. H_2_O_2_ induced oxidative stressed HUVECs (1 × 10^5^/well) were plated in a 24-well plate and incubated with BSE, CSE, and BSE-SLNPs (2 and 4 μg/ml) or vehicle control for 48 h. After incubation, cells were incubated in 400 μl of 5 μl Annexin V-FITC and 5 μl PI containing binding buffer, and then the cells were kept in the dark for 15 min at room temperature (RT). The cells were analyzed by flow cytometry (BD Biosciences, San Jose, CA) to identify the apoptotic (Annexin V positive and PI negative) and late apoptotic (Annexin V positive and PI positive) cells.

### Quantitative Polymerase Chain Reaction Analysis

Total RNA and cDNA were synthesized from vehicle control, BSE, CSE, and BSE-SLNp (4 μg/ml) treated cells using Fastlane^®^ Cell cDNA kit using qPCR. mRNA expression levels of antioxidant, proinflammatory, and tumor suppressor traits in hMSCs; adipocyte fatty acid beta-oxidation and thermogenesis (Adiponectin-R1, PPARγC1α, UCP-1, PRDM16) in adipocytes; and oxidative stress, vascular inflammation (VCAM, ICAM, EDN_1_, IL-1β, and eNOS), and vascular cell growth factor (VEGF) in HUVECs were quantified. The insulin resistance and metabolic inflammation (IL1β, IL-4, TNFα, NF-κB) related genes and the reference gene, β-actin, have been analyzed by the method of Yuan et al. (2006). The list of primer sequences is presented in Table 1. The variance has calculated the amplification values (ΔCt) between Ct (treated) and Ct (control). Gene expressions were plot using the expression of 2^−ΔΔCt^ value.

**TABLE 1:** Primers sequences used in the sybrgreen based real-time polymerase chain reaction (RT-PCR).

Primer	Forward sequence (5′ to 3′)	Reverse sequence (5′ to 3′)
LPO	CTG​CCC​TAT​GAC​AGC​AAG​AAG​C	CGG​TTA​TGC​TCG​CGG​AGA​AAG​A
GPX	GTG​CTC​GGC​TTC​CCG​TGC​AAC	CTC​GAA​GAG​CAT​GAA​GTT​GGG​C
GSK-3β	GGA​ACT​CCA​ACA​AGG​GAG​CA	TTC​GGG​GTC​GGA​AGA​CCT​T
CYP1A	GCT​GAC​TTC​ATC​CCT​ATT​CTT​CG	TTT​TGT​AGT​GCT​CCT​TGA​CCA​TCT
IL-1β	CCA​CAG​ACC​TTC​CAG​GAG​AAT​G	GTG​CAG​TTC​AGT​GAT​CGT​ACA​GG
IL-4	CCG​TAA​CAG​ACA​TCT​TTG​CTG​CC	GAG​TGT​CCT​TCT​CAT​GGT​GGC​T
NF-Kb	GCG​CTT​CTC​TGC​CTT​CCT​TA	TCT​TCA​GGT​TTG​ATG​CCC​CC
TNF-α	CTC​TTC​TGC​CTG​CTG​CAC​TTT​G	ATG​GGC​TAC​AGG​CTT​GTC​ACT​C
p53	CCT​CAG​CAT​CTT​ATC​CGA​GTG​G	TGG​ATG​GTG​GTA​CAG​TCA​GAG​C
PRb_2_	CTC​GTG​CTG​ATG​CTA​CTG​AGG​A	GGT​CGG​CGC​AGT​TGG​GCT​CC
Cdkn-2A	CCT​TCC​AAT​GAC​TCC​CTC​C	TCA​GAA​ACC​CTA​GTT​CAA​AGG​A
C/EBPα	CCGGGAGAACTCTAACTC	GATGTAGGCGCTGATGT
PPARγ	TCA​TAA​TGC​CAT​CAG​GTT​TG	CTG​GTC​GAT​ATC​ACT​GGA​G
LPL	AGGACCCCTGAAGACAG	GGCACCCAACTCTCATA
HSL	CCTCATGGCTCAACTCC	GGT​TCT​TGA​CTA​TGG​GTG​A
PPARγC_1_α	CCC​TGC​CAT​TGT​TAA​GAC​C	TGC​TGC​TGT​TCC​TGT​TTT​C
Adiponectin	CTACTGTTGCAAGCTCTC C	CTT​CAC​ATC​TTT​CAT​GTA​CAC​C
UCP-1	AGG​CTT​CCA​GTA​CCA​TTA​GGT	CTG​AGT​GAG​GCA​AAG​CTG​ATT​T
PRDM16	CCCCACATTCCGCTGTGA	CTC​GCA​ATC​CTT​GCA​CTC​A
VCAM	GAT​TCT​GTG​CCC​ACA​GTA​AGG​C	TGG​TCA​CAG​AGC​CAC​CTT​CTT​G
ICAM	AGC​GGC​TGA​CGT​GTG​CAG​TAA​T	TCT​GAG​ACC​TCT​GGC​TTC​GTC​A
EDN_1_	CTA​CTT​CTG​CCA​CCT​GGA​CAT​C	TCA​CGG​TCT​GTT​GCC​TTT​GTG​G
eNOS	GAA​GGC​GAC​AAT​CCT​GTA​TGG​C	TGT​TCG​AGG​GAC​ACC​ACG​TCA​T
VEGF	CCT​GCA​AGA​TTC​AGG​CAC​CTA​TG	GTT​TCG​CAG​GAG​GTA​TGG​TGC​T
β - Actin	GAT​CTT​GAT​CTT​CAT​GGT​GCT​AGG	TTG​TAA​CCA​ACT​GGG​ACC​ATA​TGG

### Quantification of Protein Using ELISA

The amount of vascular cell inflammation and metabolic inflammation and fatty acid metabolism-regulating protein markers such as MCP-1 and PGE (in HUVECS) and IL-1β, TNF-α, CREB-1, and AMPK (in adipocytes) were analyzed in vehicle control and BSE-SLNp treated cells using high-sensitivity ELISA-kits (Quantikine, R&D Systems, Minneapolis, MN). This assay does not distinguish between soluble and receptor-bound proteins and thus measures the total concentration of proteins. The values were expressed as pg/mg protein for all the analyzed proteins*.*

### Statistical Analysis

All the experimental group data were statistically evaluated using SPSS/28.5 software package (IBM, New York). The experimental groups data were analyzed by one-way analysis of variance (ANOVA). Then post-hoc analysis with Tukey’s range test was carried out to compare and analyze the data within the group and between the groups. All the results were expressed as mean ± SD for six replications in each group (*n* = 6). For all the comparisons, differences were considered statistically significant at *p* ≤ 0.05 and *p* ≤ 0.001 (Kim, 2014).

## Results

### Phytochemical Profiling of Basil Seed Methanol Extract and Chia Seed Extract

GC-MS chromatogram of basil seed-methanol extract and chia seed ethyl acetate extract have been presented in Supplementary Figures S1, S2. Spectral data were compared with the NIST-11 library and identified phytochemicals and their pharmacological effect are presented in Table 2. We found 99–95% similarity as per the peak values and retention time. The identified compounds with their pharmacological activities are 2,4-dinitro-N2, N3-dipropyl-6-(trifluoromethyl)-1,3-benzenediamine was known for an agonist for β-adrenergic receptor and activate AMPK level, gibberellic acid, digitoxin (heart failure treatment), gamabufotalin (suppress COX-2 *via* Iκκb/Nf-κb signaling), colchicine (decrease blood uric acid level), belcomethasone (used as mucolyte), dehydrocholic acid, prednisone, β carotene, levodopa, tetradecanoic acid, cortisone, retinol, 2-deoxy guanosine, triaziquone, phenylmercuric salicylate, prednisone, retinyl acetate, and vincamine. Some of them are known for their biological activity, whereas a few compounds remained unexplored.

**TABLE 2:** GC-MS phytochemical profiling of basil seed methanol extract (BSME) showing 99–95% similarity in the database.

Peak no	List of compounds	Molecular formula	Molecular weight	Reported biological activity
1	Gibberellic acid	C_19_H_12_O_6_	346	Anti-inflammatory Reihill et al. (2016)
2	2,4-dinitro-N2,3-dipropyl-6-(trifl uromethyl)-1,3-Benzenediamine	C_13_H_17_F_3_N_4_O_4_	350	Activate AMPK level Bung et al. (2018)
3	Digitoxin	C_14_H_16_O_13_	764	Reduces atherosclerosis Shi et al. (2016)
4	Ricinoleic acid	C_18_H_34_O_3_	298	Anti-inflammatory Vieira et al. (2001)
5	Gamabufotalin	C_24_H_34_O_5_	402	Anti-inflammatory Yu et al. (2014)
6	11-hydroxy, (11α)-Pregn-4-ene-3, 20-dione	C_21_H_30_O_3_	330	No report
7	Colchicine	C_22_H_25_NO_6_	399	Anti-inflammatory Ben-Chetrit et al. (2006)
8	Beclomethasone	C_22_H_29_CIO_5_	408	Anti-inflammatory Robroeks et al. (2008)
9	Prednisone	C_21_H_26_O_5_	358	Anti-inflammatory Yan et al. (2015)
10	ß Carotene	C_40_H_56_	536	Antioxidant and anti-inflammatory Ciccone et al. (2013)
11	Levodopa	C_9_H_11_NO_4_	197	Anti-inflammatory Maccarrone et al. (2003)
12	Tetradecanoic acid	C_28_H_56_O_2_	424	Anti-viral effect Juárez-Rodríguez et al. (2021)
13	Retinol	C_20_H_30_O	286	Antiobesity Botella-Carretero et al. (2010)
14	2-deoxy-guanosine	C_10_H_13_N_5_O_4_	267	No report
15	Triaziquone	C_12_H_13_N_3_O_2_	231	Anti-inflammatory, anti-cancer Huang et al. (2009)
16	Phenylmercuric salicylate	C_13_H_10_HgO_3_	416	Antiadherent for bacteria Pauli et al. (1997)
17	Kepone	C_10_Cl_10_O	486	Anti-cancer Legoff et al. (2021)
18	N-acetylcolchinolmethylester	C_21_H_25_NO_5_	371	No report
19	Retinyl acetate	C_22_H_32_O_2_	328	Anti-proliferation Botella-Carretero et al. (2010)
20	Vincamine	C_21_H_26_N_2_O_3_	354	Antiaging, anti-inflammatory Fayed (2010)
21	Cholesteryl benzoate	C_34_H_50_O_2_	490	No report

The chia seed fatty acid content and compositions confirmed that 77.51% of the total component was omega 3 and omega 6 fatty acids (Supplementary Figure S2). From that, 56.16% of omega 3 fatty acid and 21.35% of omega 6 fatty acid have been determined in chia seed, having been reported in our previous study (Alshatwi and Subash-Babu, 2018). The percentage area and availability of omega 3 and omega 6 have been identified as a 3:1 ratio in chia seed, where it was more beneficial for immunoregulation in circulatory immune cells (Alshatwi and Subash-Babu, 2018).

### Characterization of BSE-SLNp

**FIGURE 1:**
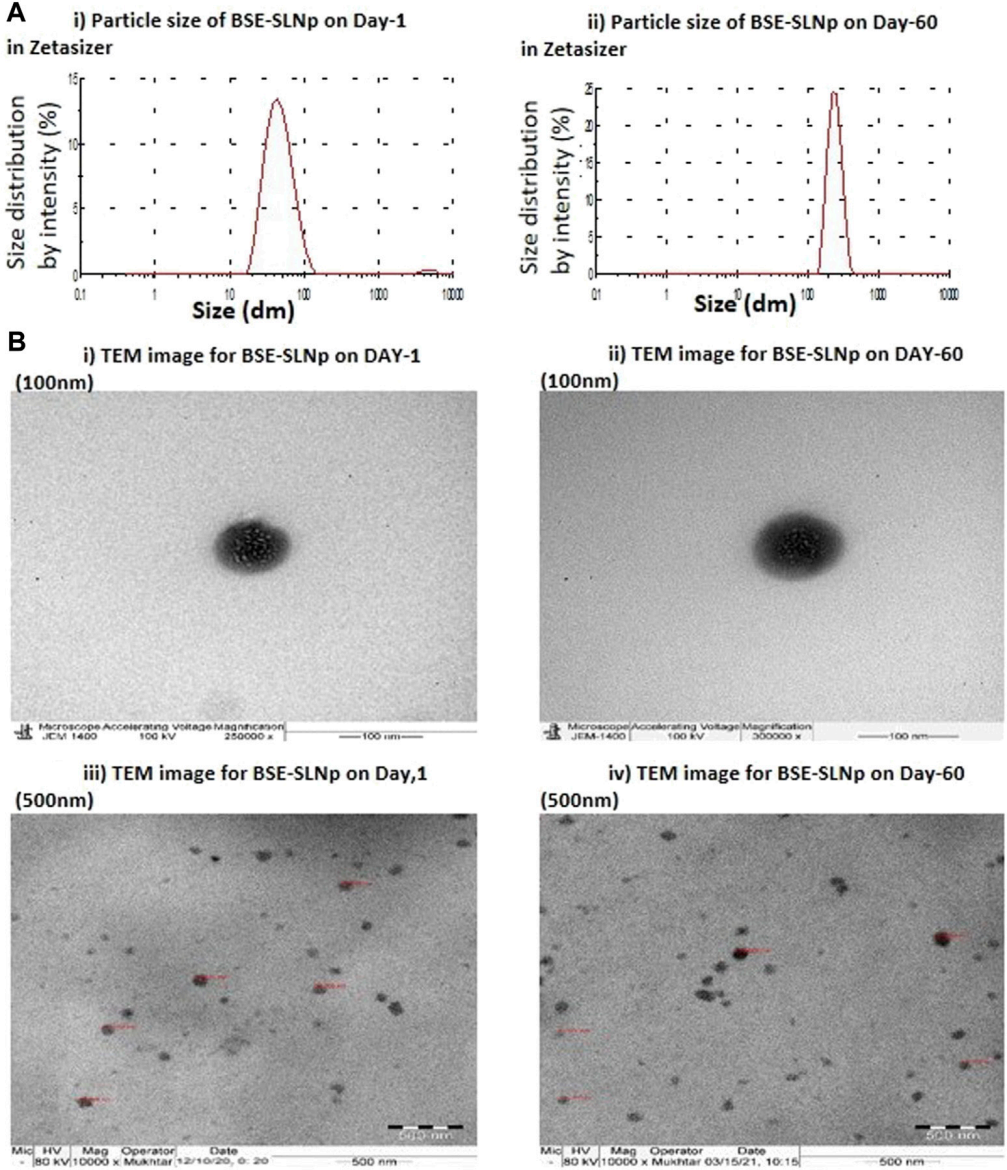
Particle size for basil seed extract (BSE) and basil seed extract loaded solid lipid nanoparticle (BSE-SLNp) on Day 1 **(1Ai)** and Day 60 **(1Aii)**. Transmission electron microscopic (TEM) image with 100-nm sale **(1Bi, ii)** and 500-nm scale **(1Biii, iv)** on Day 1 **(1Bi, iii)** and Day 60 **(1Bii, iv)**.

The efficiency of the method used in the present SLNp preparation method shows that the 80% of drug entrapment after quantified the free BSE and encapsulated BSE fractions. Encapsulation efficiency was confirmed by FT-IR data comparison between BSE and BSE-SLNp confirmed that no significant losses during formulation (Supplementary Figures S3A,B). We found the average particle size was 20–110 nm in Zetasizer analysis on preparation day 1 (Figures 1Ai,Bi,iii). The size and encapsulation remain the same even after 2 months of storage, which have been identified with an average 186 nm range peak in a Zetasizer histogram (Figure 1Aii). TEM observation confirmed that the size of individual particles remained the same in 60–105 nm (Figures 1Bii,iv). We found uniform dispersion and spherical shape of BSE-SLNp on Day 1 (Figure 1Biii) and after storage (Figure 1Biv).

### Effective Dose Determination

The results of effective dose determination analysis confirmed that the tested 2, 4, and 8 μg/ml doses of BSE-SLNp have not shown any toxicity-associated morphological changes in hMSCs, preadipocytes, or HUVECs. Mitochondrial membrane potential (MMP, JC-1) has predicted the metabolic active cells having the capacity of oxidative potential and energy metabolism in cells. In the present study, 4 and 8 μg/ml doses show high J-aggregates (accumulated red color) representing metabolically active cells with healthy mitochondria and high mitochondrial membrane potential compared to 2 μg/ml dose in hMSCs, preadipocytes, and HUVECs (Supplementary Figure S4). A 2-μg/ml dose was found with improved mitochondrial membrane potential but comparatively lower than 4 and 8 μg/ml. Based on the above findings, 4 μg/ml have been selected as an effective dose for further molecular analysis, also the dose is found to be safe therapeutically. There was a 4-μg/ml dose used for BSE and CSE also to compare the beneficial effect.

### Biosafety of BSE-SLNp in hMSCs

#### Cytotoxicity, Nuclear Morphology, and JC-1 Staining Analysis in hMSCs

**FIGURE 2:**
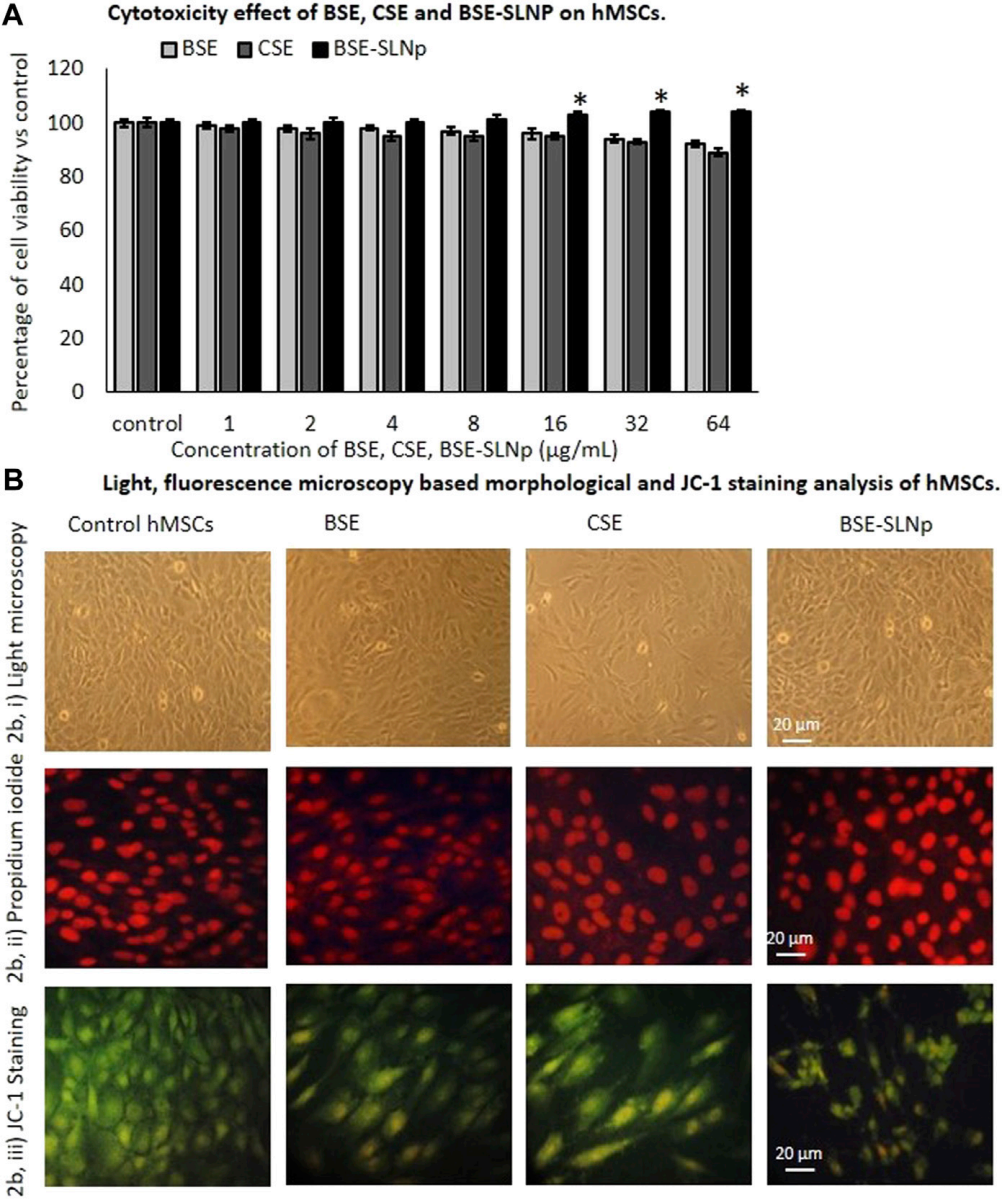
*In vitro* cytotoxicity **(2A)**, light microscopic **(2Bi)**, propidium iodide **(2Bii)**, and JC-1 staining **(2Biii)** florescence microscopic image analysis of vehicle control, BSE, CSE, and BSE-SLNp treated human mesenchymal stem cells after 48 h. Each value is means ± SD (*n* = 6). *p ≤ 0.05 by comparison with vehicle control. In PI staining, the nucleus appeared to be normal and there are no signs of shrunken, pyknosis, or apoptotic nucleus. JC-1 fluorescence images showing merged images of the red and green signals of the dye, corresponding to JC-1 in J-aggregates vs. monomeric form. We found less J-aggregates and hypertrophic adipocyte in control cells. In BSE-SLNp treated adipocyte showing high J-aggregates directly representing (high MMP, Δψ_m_) active mitochondria compared to BSE or CSE.

We used freshly prepared BSE, CSE, and BSE-SLNp for the cellular and molecular biology assays. The cytotoxicity assay confirmed that a 4-µg/ml concentration of BSE and CSE reduced the hMSCs cell proliferation with a non-significant range of 8 and 11%, respectively. But, BSE-SLNp treatment increased the hMSC’s growth up to 4% within 48 h (Shown in Figure 2A). In addition, light microscopy and florescent PI staining of experimental cells show normal morphology without any irregular shape or condensed nuclear materials (Figure 2Bii). Mitochondrial membrane potential (MMP, JC-1) has predicted the oxidative capacity, energy production, and energy metabolism levels in experimental cells. JC-1 staining results also show the highest mitochondrial membrane potential representing the accumulated red color in BSE-SLNp treated cells. Compared to BSE and CSE, the BSE-SLNp showed significantly increased J-aggregates (Figure 2Biii).

#### Quantification of Gene Expression Levels in hMSCs

**FIGURE 3:**
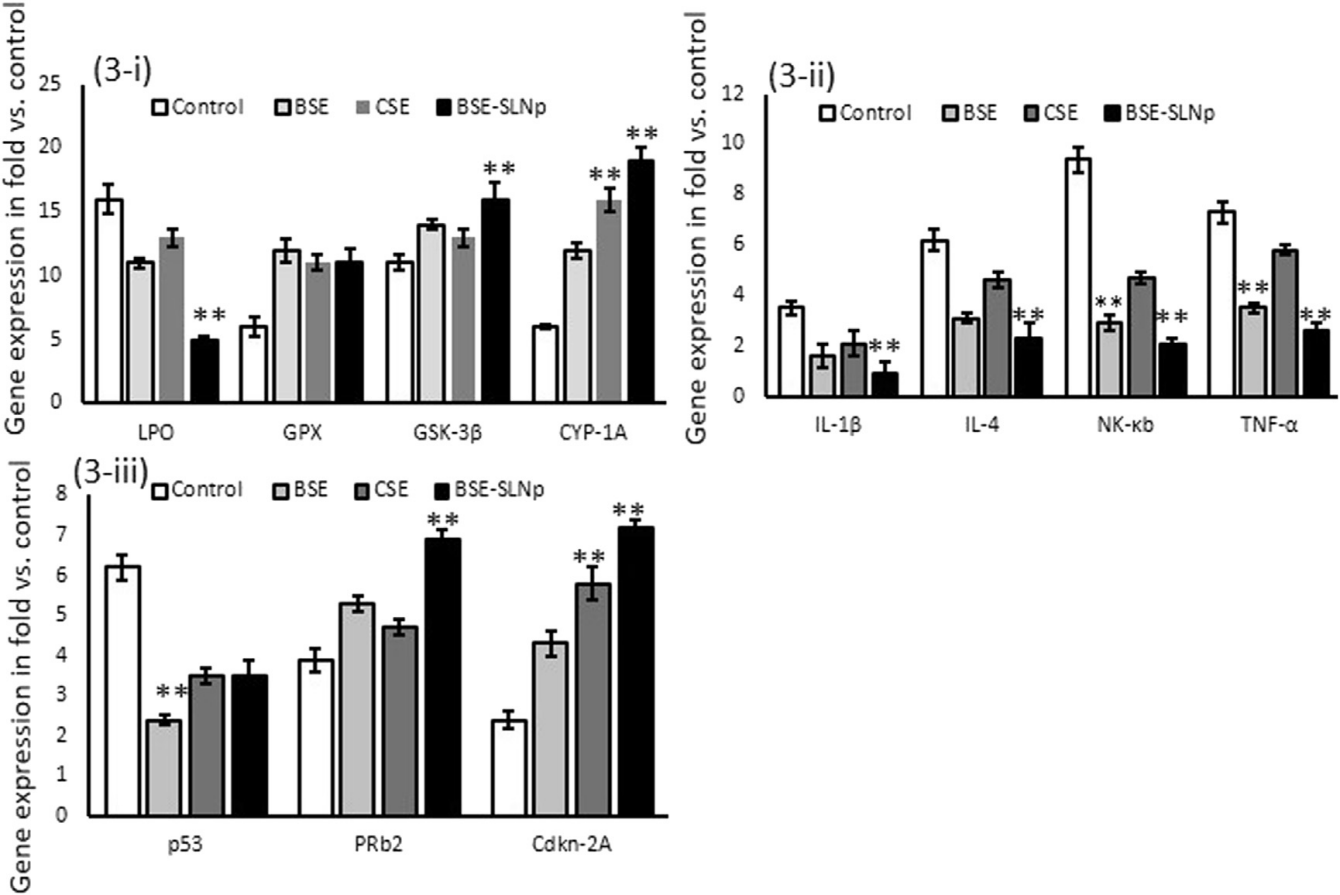
Effect of BSE, CSE, and BSE-SLNp on oxidative stress **(3i)**, proinflammatory **(3ii)**, and tumor suppressor **(3iii)** related gene expression levels in hMSCs after 48 h. Each value is means ± SD (*n* = 6). **p ≤ 0.001 by comparison with vehicle control, BSE, and CSE.

Antioxidant (LPO, GPX, GSK-3β, CYP1A), proinflammatory (IL1β, IL-4, NF-kB, and TNF-α), and tumor suppressor (p53, PRb_2_, and Cdkn-2A) related gene expression levels were quantified in vehicle control, BSE, CSE, and BSE-SLNp treated hMSCs after 48 h (Figure 3). Observed results confirmed that the mRNA expression levels LPO, IL-1β, IL-4, NF-kB, and TNF-α levels significantly (*p* ≤ 0.001) decreased and increased PRb_2_ and Cdkn-2A expressions. There were no significant alterations in the BSE-SLNp treated p53 expression levels when compared to vehicle control.

### Effect of BSE-SLNp Against Preadipocytes Maturation

#### Cytotoxicity, Lipid Accumulation Analysis, and JC-1 Staining Analysis in hMSCs

**FIGURE 4:**
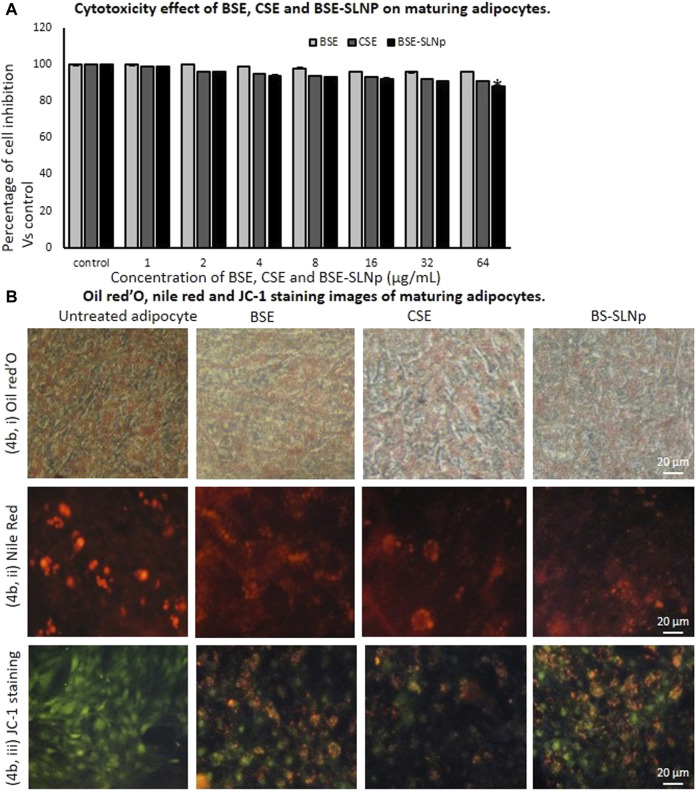
*In vitro* cytotoxicity after 48 h **(4A)**, Oil Red O **(4Bi)**, Nile red **(4Bii)**, and JC-1 **(4Biii)** stained microscopic image analysis of vehicle control, BSE, CSE, and BSE-SLNp treated maturing adipocyte after 14 days. Each value is means ± SD (*n* = 6). *p ≤ 0.05 by comparison with vehicle control. In Oil Red O staining and Nile red staining, vehicle control showing hypertrophic adipocyte directly propositional to triglyceride storage. But in BSE-SLNp treatment shown controlled adipocyte maturation, less lipid accumulation, and less florescent staining. BSE-SLNp treated cells showing the highest reduction compared to BSE or CSE. JC-1 fluorescence images showing merged images of the red and green signals of the dye, corresponding to JC-1 in J-aggregates vs. monomeric form. We found less J-aggregates and hypertrophic adipocyte in vehicle control. In BSE-SLNp treated adipocyte showing high J-aggregates directly representing active mitochondria (high MMP, Δψ_m_).

In preadipocytes, BSE, CSE, or BSE-SLNp did not produce significant cytotoxicity. As shown in Figure 4A, BSE, CSE, or BSE-SLNp treatment produced minimal cell growth inhibition such as 6, 9, and 12%, respectively. Figures 4Bi,ii shows the Oil Red O and Nile red staining to determine lipid accumulation levels in maturing adipocytes after 14 days of BSE-SLNp treatment. We found an 85% reduction of lipid levels when compared to vehicle control. Most notable, BSE or CSE treatment show the morphology of hypertrophic adipocytes, but BSE-SLNp treatment shows smaller lipid droplets with reduced lipid accumulation (brown adipocyte morphology).

Adipocyte’s mitochondrial membrane potential (MMP, Δψm) is directly proportional to the oxidative capacity and energy metabolism level. In Figure 4Biii, BSE-SLNp treated maturing adipocytes showing the images of JC-1 staining clearly represent dye having red and green signals, comparable to J-aggregates vs. monomeric form. In vehicle control, hypertrophic adipocytes with less J-aggregates confirmed less mitochondrial potential than BSE, CSE, or BSE-SLNp treated cells. Nevertheless, BSE-SLNp showing linear and spindle shaped adipocytes with high J-aggregates compared to BSE and CSE directly represents the potential of BSE-SLNp on mitochondrial efficiency with fatty acid oxidation.

#### Quantification of Gene Expression Levels in Maturing Adipocytes

**FIGURE 5:**
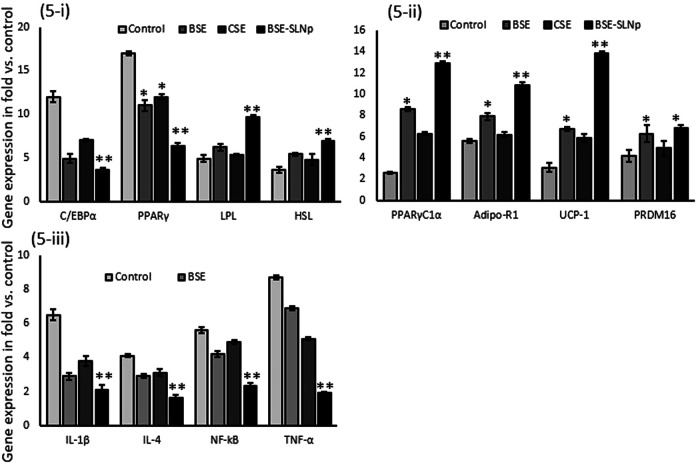
Effect of BSE, CSE, and BSE-SLNp on adipogenic **(5i)**, mitochondrial thermogenesis **(5ii)**, and metabolic inflammation **(5iii)** related gene expression levels in maturing adipocyte after 14 days. Each value is means ± SD (*n* = 6). *p ≤ 0.05 by comparison with vehicle control. **p ≤ 0.001 by comparison with vehicle control, BSE, and CSE.

In BSE-SLNp treated maturing adipocytes, mRNA expression levels C/EBPα, PPARγ were significantly decreased and lipoprotein lipase (LPL), hormone-sensitive lipase (HSL) expression were increased when compared to cells treated with BSE, CSE, or vehicle control. Most interestingly, we found significantly increased mRNA expression levels of PPARγC_1_α, adiponectin-R1, UCP-1, and PRDM16, presented in Figure 5. The metabolic inflammation-related genes such as IL-1β, IL-4, NF-kB, and TNF-α expressions were decreased in BSE-SLNp treated maturing adipocytes. Overall, BSE-SLNp showed a highly significant effect when compared to BSE or CSE-treated cells.

### Effect of BSE-SLNp in HUVECs

#### Cytotoxicity, Microtubule Development, Cell and Nuclear Morphology, and JC-1 Staining Analysis in HUVECs

**FIGURE 6:**
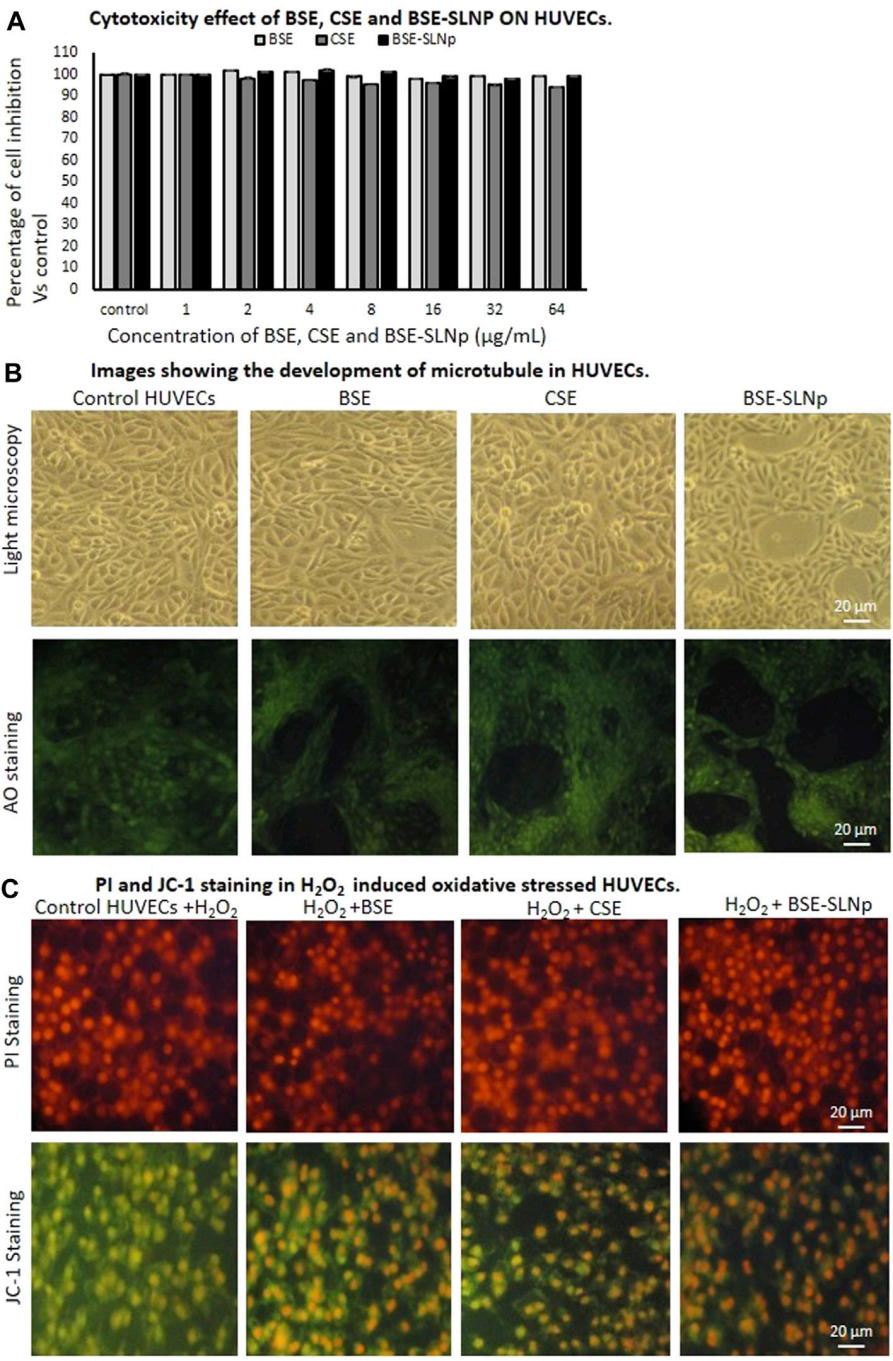
*In vitro* cytotoxicity **(6A)**, microtubule development **(6B)**, PI and JC-1 **(6C)** stained microscopic image analysis of vehicle control, BSE, CSE, and BSE-SLNp treated H_2_O_2_ induced oxidative stressed HUVECs after 48 h. Each value is means ± SD (*n* = 6). In PI staining, the nucleus appeared to be normal and there are no signs of shrunken, pyknosis, or apoptotic nucleus. BSE-SLNp treated adipocyte showing high J-aggregates directly representing active mitochondria (high MMP, Δψ_m_).

*In vitro* cytotoxic effects of BSE, CSE, and BSE-SLNp against HUVECs are presented in Figure 6A, and there were no significant cell inhibitions observed in all the treatment compared to vehicle control. Figure 6B shows the microtubule development morphology under light microscopic and florescence microscopic (acridine orange) images. In Figure 6C, the PI staining of H_2_O_2_ induced oxidative stressed HUVECs treated with BSE-SLNp shows spherical shaped nucleus without any nuclear condensation compared to BSE and CSE. JC-1 staining of BSE-SLNp treated to H_2_O_2_ induced oxidative stressed HUVECs confirmed that 89.4% of negatively charged mitochondria converted the lipophilic cationic JC-1 (green colored) to red colored J-aggregates when compared to BSE (82.8%) or CSE (65%, data not shown) treatments. BSE or CSE treatments showed significantly higher J-aggregates compared to vehicle control.

#### FACS Assisted Mitochondrial Membrane Potential (Δψm) (BD Mito Scan) and Annexin-v/Apoptosis Analysis in HUVECs

**FIGURE 7:**
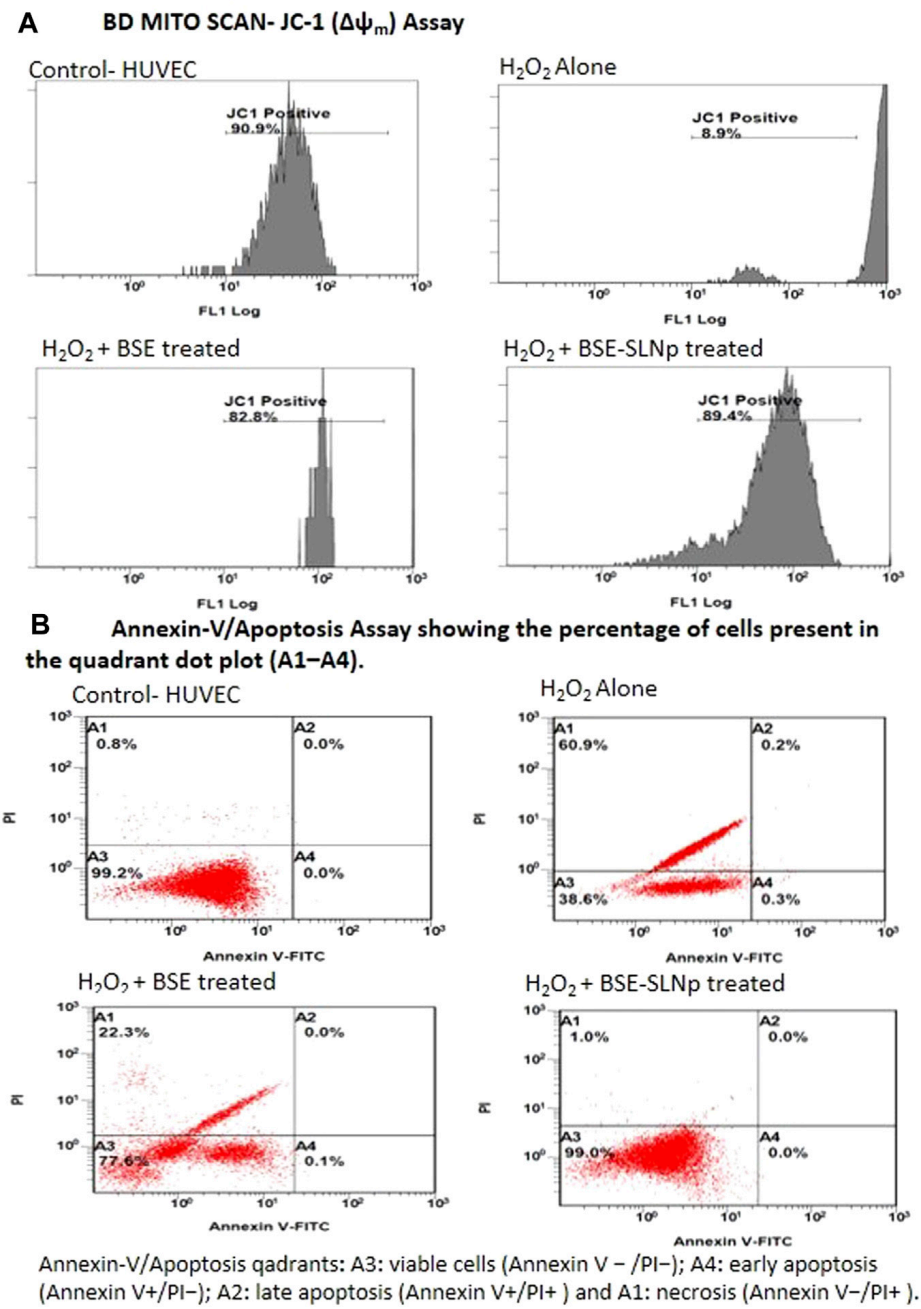
Flow cytometry analysis of JC-1 (Δψ_m_, BD Mito Scan) and annexin-V/propidium iodide double staining in H_2_O_2_ induced oxidative stressed HUVECs treated with BSE and BSE-SLNp. Untreated cells were considered a negative control, whereas H_2_O_2_ (40 mM) was added for positive control.

Figure 7A shows the mitochondrial membrane potential capacity of BD Mito Scan analysis after BSE, BSE-SLNP treated H_2_O_2_ induced oxidative stressed HUVECs. BSE-SLNp treatment increased MMP (Δψm) to 89.4 ± 5.2% when compared to H_2_O_2_ alone treated HUVECs (9.5 ± 3.7%). BSE alone treated cells showed 82.8 ± 3.9% percentage of increased MMP (Δψm) compared to H_2_O_2_ alone treated HUVECs.

As shown in Figure 7B, the quadrants representing annexin-V assays are A1-necrosis, A2-late apoptosis, A3-viable cells, and A4-early apoptosis. H_2_O_2_ alone treated HUVECs show 38.6 ± 5.1% of viable cells (A3), 0.5 ± 0.01% in apoptotic (A2 and A4), and 60.9 ± 3.4% in necrosis quadrants (A1). However, BSE-SLNp treated oxidative stressed HUVECs appeared 99.0 ± 0.7% cells in the viable cell (A3) quadrant and 1 ± 0.2% in the necrosis (A1) quadrant. In the meantime, BSE alone treated HUVECs show 77.6 ± 2.8% cells in viable cells and 22.3 ± 3.8% in necrotic quadrants. Observed results indicated that BSE treatment decreased HUVEC necrosis up to 38% when compared to H_2_O_2_ alone treated HUVEC. Overall, BSE-SLNp highly (99%) protects the HUVECs from H_2_O_2_ induced oxidative stress.

#### Quantification of Gene Expression Levels in HUVECs

**FIGURE 8:**
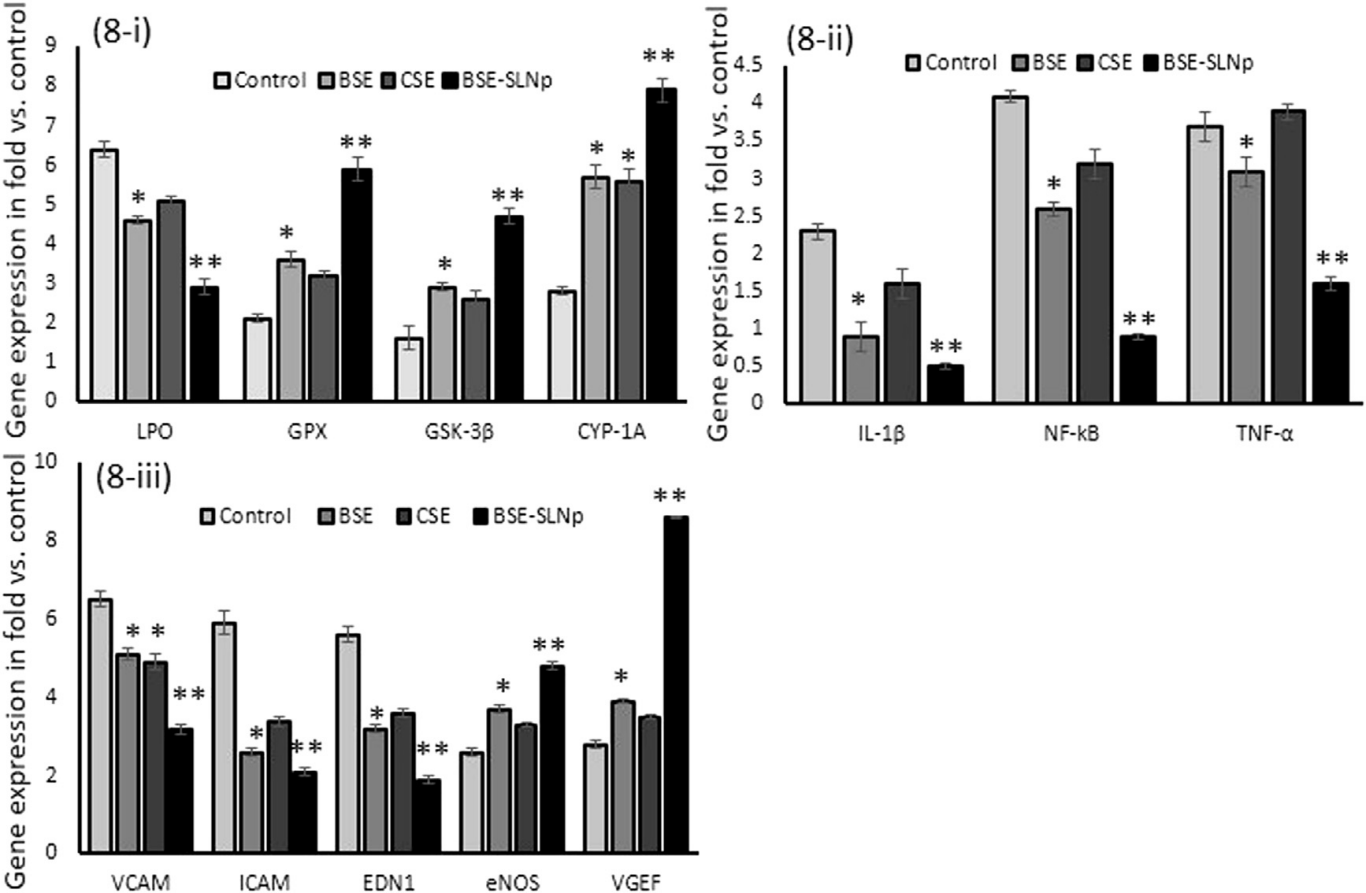
Effect of BSE, CSE, and BSE-SLNp on oxidative stress and antioxidant **(8i)**, proinflammatory **(8ii)**, and vascular cell inflammation **(8iii)** related gene expression levels in H_2_O_2_ induced oxidative stressed HUVECs after 48 h. Each value is means ± SD (*n* = 6). *p ≤ 0.05 by comparison with vehicle control. **p ≤ 0.001 by comparison with vehicle control, BSE, and CSE.

Gene expression levels of oxidative stress (LPO, GPX, GSK-3β, and CYP1A) and vascular inflammation (VCAM, ICAM, EDN_1_, eNOS, NF-kB, IL-1β, and TNF-α) and vascular endothelial cell growth factor (VEGF) have been quantified in vehicle control, BSE, CSE, and BSE-SLNp treated HUVECs after 48 h (Figure 8). We found significantly (*p* ≤ 0.001) increased levels of LPO, VCAM, eNOS, ICAM, NF-kB, IL-1β, and TNF-α expression levels in H_2_O_2_ induced HUVECs. However, HUVECs treated with BSE-SLNp and H_2_O_2_ significantly decreased the vascular inflammation and oxidative stress-related mRNA expression than BSE or CSE treatment. Most interestingly, VEGF expression levels have been significantly increased to twofold in BSE-SLNp treated cells compared to BSE or CSE treatment. VEGF expression was not detected in H_2_O_2_ induced oxidative stressed HUVECs.

#### Quantification of Protein Levels Using the ELISA Method

**FIGURE 9:**
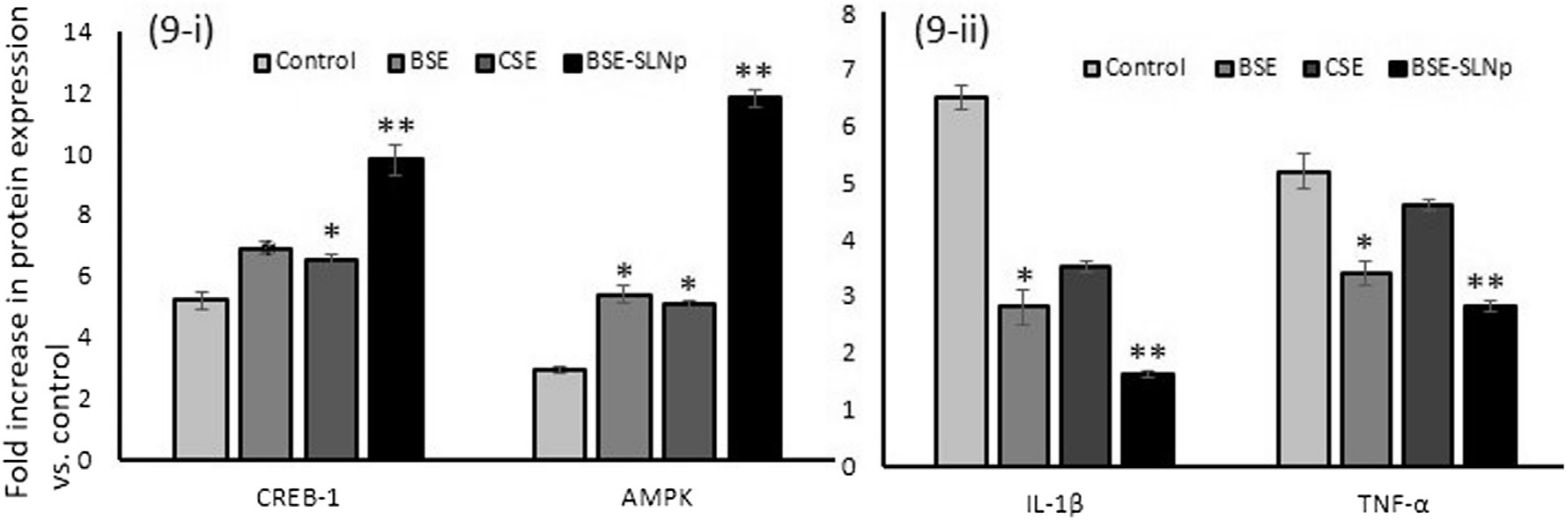
Quantification of adipocyte energy metabolism **(9i)** and vascular cell inflammation **(9ii)** related protein levels in BSE, CSE, and BSE-SLNp treated maturing adipocyte (14 days) and HUVECs (2 days) using the ELISA method. Each value is means ± SD (*n* = 6). *p ≤ 0.05 by comparison with vehicle control. **p ≤ 0.001 by comparison with vehicle control, BSE, and CSE.

Fatty acid metabolism-regulating markers, such as CREB-1 and AMPK (adipocytes), and metabolic vascular cell inflammatory markers, such as IL-1β and TNF-α (HUVECs), were analyzed in vehicle control and BSE-SLNp treated cells. As shown in Figure 9i, in maturing adipocytes, we found significantly (*p* ≤ 0.001) increased levels of CREBp-1 (twofold) and AMPK (fourfold) when compared to vehicle control or BSE or CSE treatment. The results presented in Figure 9ii show that the protein levels of IL-1β (twofold) and TNF-α (1.5-fold) were significantly decreased in BSE-SLNp treated cells when compared to vehicle control, BSE, or CSE.

## Discussion

hMSCs are majorly applied in translational research and regenerative medicine because of their higher plasticity and they have been characterized by their trans-differentiation, and paracrine and immunosuppressive properties (Castro-Manrreza and Montesinos, 2015; Denu and Hematti, 2016). Cells can generally protect themselves from ROS damage through their self-defense antioxidative mechanisms. Meanwhile, hMSCs have a lesser antioxidant capacity and are more sensitive to oxidative stress when compared to differentiated lineages such as adipocytes, chondrocytes, and osteoblast (Yagi et al., 2013). In regenerative medicine, cellular stress produces excessive ROS or exogenous stress-induced free radical might impair the capacity of differentiation to multiline ages or self-renewal and proliferation (Zou et al., 2004). Excessive free radical damage to hMSCs might end with cell senescence and arrested cell divisions (Bajek et al., 2012). ROS are initially generated from the mitochondrial complex (I and III) and NOX4 during hMSC’s differentiation (Devine et al., 2001). Excessive ROS react and damage the biomolecules, significantly altering the integrity of genomic DNA, which is critical for cellular proliferation and functions (Kobayashi and Suda, 2012). Excessive ROS induces mitochondrial dysfunction, resulting in abnormal mitochondria dynamics, altered gene expression, and enzyme activities (Mao and Reddy, 2010). Intracellular ROS undergoes oxidation reaction and interacts with biological molecules such as DNA, RNA, proteins, or peroxidation of lipids. Orciani et al. (2010) reported that ROS inhibits hMSCs to osteogenesis differentiation, resulting in bone weakness and arthritis.

Dietary habits regulate the mitochondrial metabolic state, which determines the physiological rate of the mitochondrial production of O2^•−^ and H_2_O_2_ associated with the electron transfer chain. Excessive reactive oxygen species causes mitochondrial components damage and initiates functional degradation, which forms the central dogma of “The Free Radical Theory of Inflammation, Obesity and Aging” (Cadenas and Davies, 2000). Developing phyto-remedies with intracellular or inner mitochondrial stimulation to regulate oxidative-reduction in hMSCs or β-oxidation of fatty acids in adipocytes became very challenging because the low intracellular uptake of bioactive compounds ends with low biological availability and loss of efficiency. Uptake of the bioactive compound across the cellular lipid bilayer without any conformational change might provide maximum efficiency and deserve their application in mitochondrial oxidative reduction regulation. SLNp is most favorable in facilitating effective diffusion through the cell membrane and increasing active ingredient’s bioavailability.

SLNp consists of better biocompatibility and biodegradability and tends to incorporate hydrophilic and hydrophobic compounds (Dolatabadi et al., 2014). SLNp is most suitable because of its less solvent requirement and efficient surfactant-based water/oil/water combinations with the stability of nanoparticles for a longer duration in the room or at body temperature (Jain et al., 2014). Basil seed-loaded solid lipid nanoparticles have been fabricated using organic chia seed as the encapsulating liposome material, which was achieved with nanometer size, equally dispersed, and less aggregation capacity. A dose determination study confirmed that 4 μg/ml of BSE-SLNp was an effective dose which achieved non-toxic and metabolically active cells in the tested cell models. The biosafety of BSE-SLNp has been established in hMSCs, the safety and nontoxic character of nanoparticles have been confirmed by the increased cell viability, normal morphology with the spherical shaped nucleus. BSE-SLNp treated cells confirmed the reduced cellular stress and increased mitochondrial membrane potential (ψ_m_) compared to the BSE or CSE. Most interestingly, the levels of oxidative lipid peroxide (LPO) and tumor suppressor p53 expressions have been decreased. In addition, proinflammatory genes such as IL1β, IL-4, NF-kB, and TNF-α levels have been decreased in BSE-SLNp treated hMSCs. Maintenance of cellular integrity and nuclear replication stability by the BSE-SLNp has been confirmed by relatively increased mRNA expressions of PRb_2_ and Cdkn-2A. Stem cell therapies for nervous system disorders have revealed promising results on targeting and downregulating the proinflammatory factors such as IL-1β, TNF-α, and IFN-γ (Rad et al., 2019). Di Nicola et al. (2002) have reported that hMSCs implanted to injured tissues and contributed to tissue repair after suppressing inflammatory rejection. In this context, ferulic acids, quercetin, and curcuminoid have been developed as an SLNp and confirmed their efficiency in *in vitro*, *in vivo* systems and as a human dermal cream application without producing side effects (Plianbangchang et al., 2013; Borges et al., 2020).

In developed countries, most people’s life spans and health spans have the consequences of cardiovascular complications, especially relevant to the aging or age-related alterations. High triglyceride accumulation in white adipose tissue (WAT) associated with excessive nutrient intake and less energy expenditure ends with visceral obesity. Visceral obesity initiates WAT dysfunction, and it is considered an important hallmark of the aging process, further contributing to metabolic alterations and systemic proinflammatory conditions and multi-organ damage (Pérez et al., 2016). BSE-SLNp material effectively arrested the lipid accumulation and stimulated fatty acid beta-oxidation, evidenced by high MMP (Δψm) of active mitochondria in maturing adipocytes.

The lipolytic effect of BSE-SLNp might be due to the bioactive principle, 2,4-dinitro-N2,N3-dipropyl-6-(trifluoromethyl)-1,3-benzenediamine and gibberellic acid present in BSE, which is the stimulator of the β-adrenergic receptor followed by the activation of AMPK, effectively increasing the protein expression levels of CREBp-1 and AMPK. In this context, Bung et al. (2018) have established that 3, 2-[2-(4-(trifluoromethyl)phenylamino) thiazol-4-yl] acetic acid was effective as an AMP mimetic or AMPK activator *in vitro* and *in vivo*. cAMP pathways and AMP-activated protein kinase (AMPK) were recognized as cellular energy sensors vital for the reserve of adipocyte maturation (Zhang et al., 2015). C/EBP-α is a central transcriptional adipogenic activator regulated by cAMP-responsive element-binding protein (CREB). cAMP binds to the regulatory subunit PKA and release the catalytic subunit; it phosphorylates the lipid metabolism-associated protein substrates, including AMPK, in peripheral and subcutaneous adipocytes (Moon et al., 2019). AMPK activated during depletion of the cellular ATP increases the AMP/ATP ratio and initiates metabolic and genetic events to restore ATP levels *via* fatty acid beta-oxidation in adipocytes (Cheng et al., 2015). In adipocytes, during lipolysis, AMP-activated protein kinase is activated and decreases the stored lipid content *via* thermogenesis (Yin et al., 2003). Adipocyte differentiation was inhibited by phosphorylated AMPK *via* suppressing the major adipogenesis regulators, such as C/EBPα and PPARγ, the central regulators of adipogenesis and lipid store in adipocytes (Gauthier et al., 2008).

In our study, the efficiency of mitochondria was confirmed by the higher expression of energy production-related mRNAs such as PPARγC1α, adiponectin-R1, UCP-1, and PRDM1. In adipocytes, the increase of CREBp-1 activity *via* the cAMP–PKA pathway stimulates the level of PPARγC_1_α, which activates the major thermogenic genes PRDM16 and UCP_1_ (Cheng et al., 2015; Zhang et al., 2015). Also, the adipocyte hyperplasia stimulating factors, C/EBPα and PPARγ*,* have significantly decreased. The beneficial mitochondrial thermogenic potential of BSE-SLNp reverses the paradigm of adipose tissue dysfunction associated proinflammatory markers associated with the biological similaritsies of the aging process such as chronic inflammation and multi-system alterations.

Pathophysiology of vascular diseases associated with hypertrophic adipocyte followed by hyperplasia, specifically perivascular adipocyte, might play a significant role in neovascularization around fat depots (Miao and Li, 2012). Hypertrophic adipocyte secreting adipokines might provide a new link between obesity and vascular complications (Nakamura et al., 2014). Schlich et al. (2013) found that matured adipocyte secreted adipokines supplemented with oleic acid (OA) increased the vascular smooth muscle cell proliferation *via* induction of iNOS expression, NO production, and proinflammatory signaling. In the present study, BSE-SLNp treatment increased HUVECs proliferation capacity, microtubule morphology, and nuclear integration. The above observation was confirmed by an increased MMP (ψ_m_) up to 89.4% in H_2_O_2_ induced oxidative stressed HUVECs, but in vehicle control found with 9.5% of MMP (ψ_m_). In this context, da Silva et al. (2020) have identified the protective effect of beet leaves extract against oxidative stress in HUVECs. The BSE-SLNp confirmed the endothelial cell proliferation capacity, such as the highest (99%) viable cells present (A3 quadrant) in oxidative stress stimulated HUVECs. In addition, vascular endothelial cell growth factor (VEGF) expression has been found to be increased in HUVECs.

Endothelial NO^•^ synthase (eNOS) is the predominant isoform of NOS, responsible for most of the NO^•^ products in smooth muscle cells and vascular tissues. Vascular NO^•^ dilates all types of blood vessels, and protects platelet aggregation and leukocyte adhesion in endothelial cells (Forstermann et al., 1994). In the present study, the mRNA expression levels of eNOS have increased in BSE-SLNp treated H_2_O_2_ induced oxidative stressed HUVECs. Many conflicts about cardiovascular risk factors and endothelial dysfunctions are associated with decreased or increased eNOS expression (Li et al., 2002). The increased expression of eNOS in vascular disease might be due to the consequence of excessive production of H_2_O_2_. Dismutation product O_2_^•−^ can increase eNOS expression through transcriptional and post-transcriptional mechanisms (Drummond et al., 2000). Accelerated degradation of NO• has accompanied pathogenesis of vascular disease after reaction with O_2_^•−^ and finally ONOO^−^ formed, which in turn leads to eNOS uncoupling and NADPH oxidase enzyme dysfunction (Förstermann and Münzel, 2006). The oxidative stress-related mRNAs of LPO, cellular adhesion molecules VCAM and ICAM, proinflammatory agents NF-kB, IL-1β, and TNF-α expression levels significantly decreased BSE-SLNp against BSE or CSE alone treated HUVECs. In this context, Banerjee et al. (2016) have reported that treatment with paclitaxel-loaded solid lipid nanoparticles enhanced anti-angiogenic and anti-glioma therapy.

## Conclusion

The development of drugs with beneficial effects on the prevention and treatment of obesity must gain more attention with its associated metabolic syndrome. The fabricated BSE-SLNp has the bioactive principle, 2,4-dinitro-N2,N3-dipropyl-6-(trifluromethyl)-1,3-benzenediamine and gibberellic acid, which stimulate fatty acid β oxidation *via* CREBp-1, AMPK pathway *via* a β-adrenergic receptor, and effectively arrest adipocyte hypertrophy. Further, increased adipocyte’s mitochondrial energy production *via* CREBp-1, AMPK, decreased adipokine secretion and proinflammatory cytokines without inducing cellular toxicity in hMSCs or adipocytes. In addition, HUVEC’s proliferation capacity and microtubule development have been supportive of the neovascularization around hyperplastic adipocytes. BSE-SLNp has arrested oxidative stress-induced proinflammatory cytokines, and this effect might be supportive for the immunoregulatory and aging-related vascular diseases therapy. *In vivo* study will be warranted with AMPK knockout or transgenic animal models to confirm the BSE-SLNPs oral administration and efficacy of angiogenic and immunomodulatory potential.

## Data Availability

The original contributions presented in the study are included in the article/Supplementary Material, further inquiries can be directed to the corresponding author.
