# Supplementary material for: Evaluation of Biosafety, Antiobesity, and Endothelial Cells Proliferation Potential of Basil Seed Extract Loaded Organic Solid Lipid Nanoparticle
Source: Front Pharmacol. 2021 Oct 4;12:722258. doi: 10.3389/fphar.2021.722258 (PMC8521050; doi:10.3389/fphar.2021.722258)
Supplement: Supplementary file 1 [file Presentation1.PDF]

Supplementary figures.

Supplementary figure 1. Gas chromatography – mass spectrum (GC-MS) chromatogram for basil seed methanol extract.

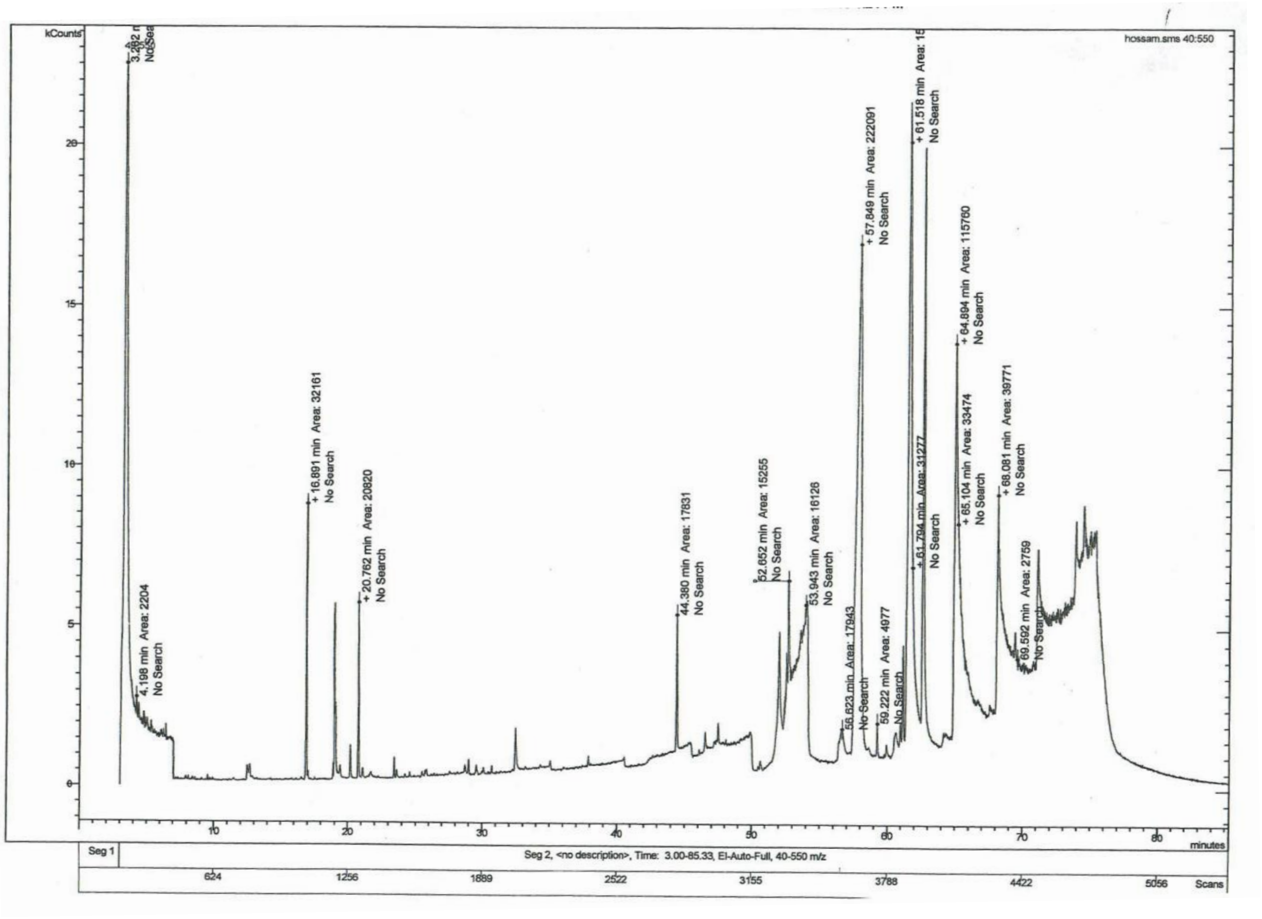

Spectral data were compared with NIST-11 library to identify the phytochemicals. We found 99%-95% similarity as per the peak values and retention time.

Supplementary figure 2. Gas chromatography – mass spectrum (GC-MS) chromatogram for chia seed methanol extract.

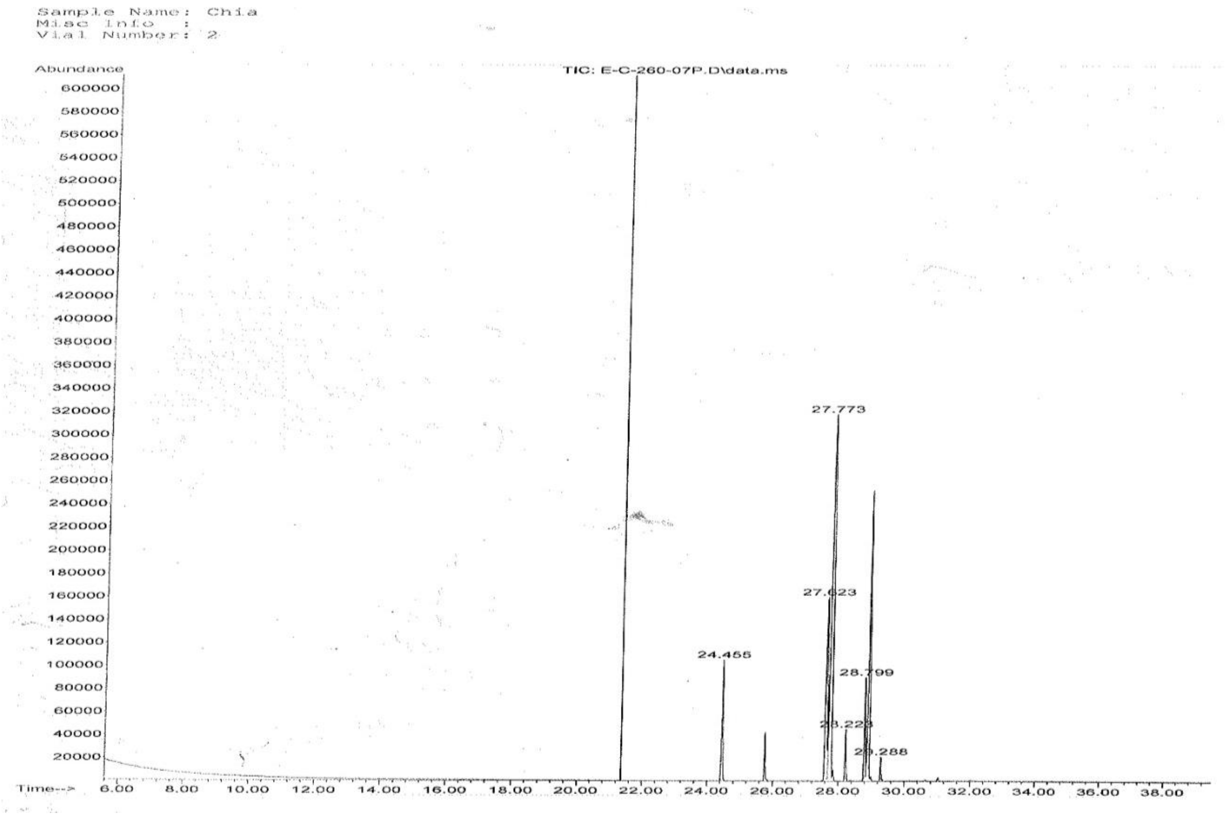

Spectral data were compared with NIST-11 library to identify the phytochemicals. We found 99%-95% similarity as per the peak values and retention time.

Supplementary figure 3. Comparative FT-IR spectra analysis between basil seed extract (BSE) (3a) and basil seed solid lipid nanoparticles (BSE-SLNp) (3b).

Supplementary figure - 3a, FT-IR spectra for basil seed extract

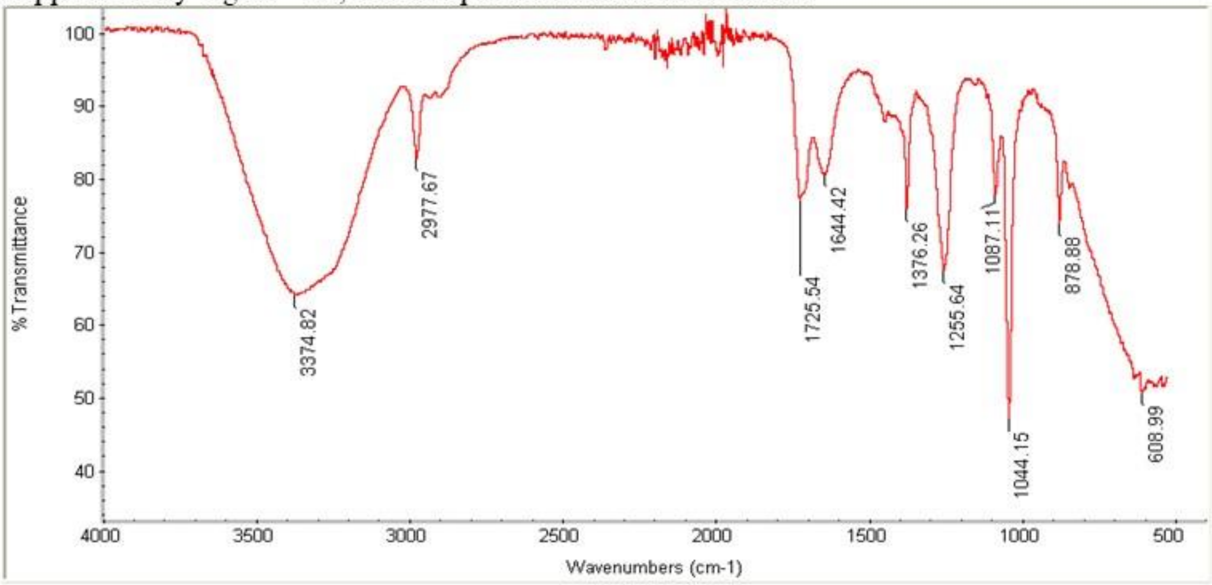

Supplementary figure – 3b, FT-IR spectra for BSE-SLNp

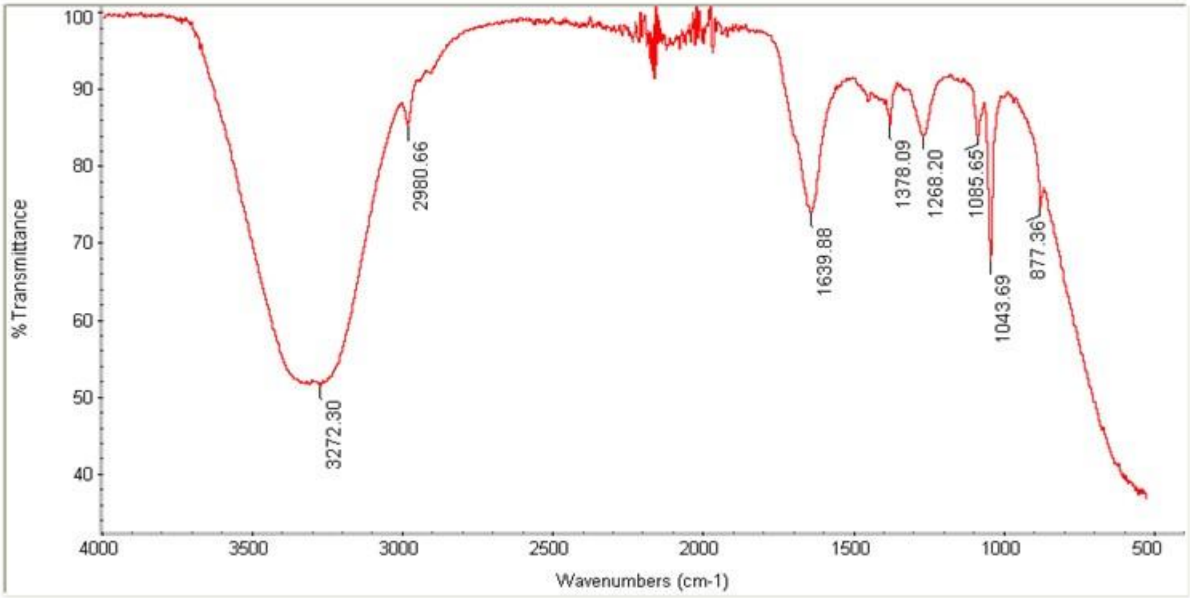

FT-IR data used to identify the encapsulation efficiency of basil seed extract (BSE) during the formulation of solid lipid nanoparticles (BSE-SLNp), the results confirmed that no significant losses during SLNp formulation.

Supplementary figure 4. Effective dose determination of BSE-SLNp in human mesenchymal stem cells (hMSCs), adipocytes and human umbilical vein endothelial cells (HUVECs) after 48 hr of vehicle control, 2 μg/mL, 4 μg/mL and 8 μg/mL of BSE-SLNp treatment. Effective dose have been identified by increased mitochondrial membrane potential using JC-1 staining representing the metabolically active cells.

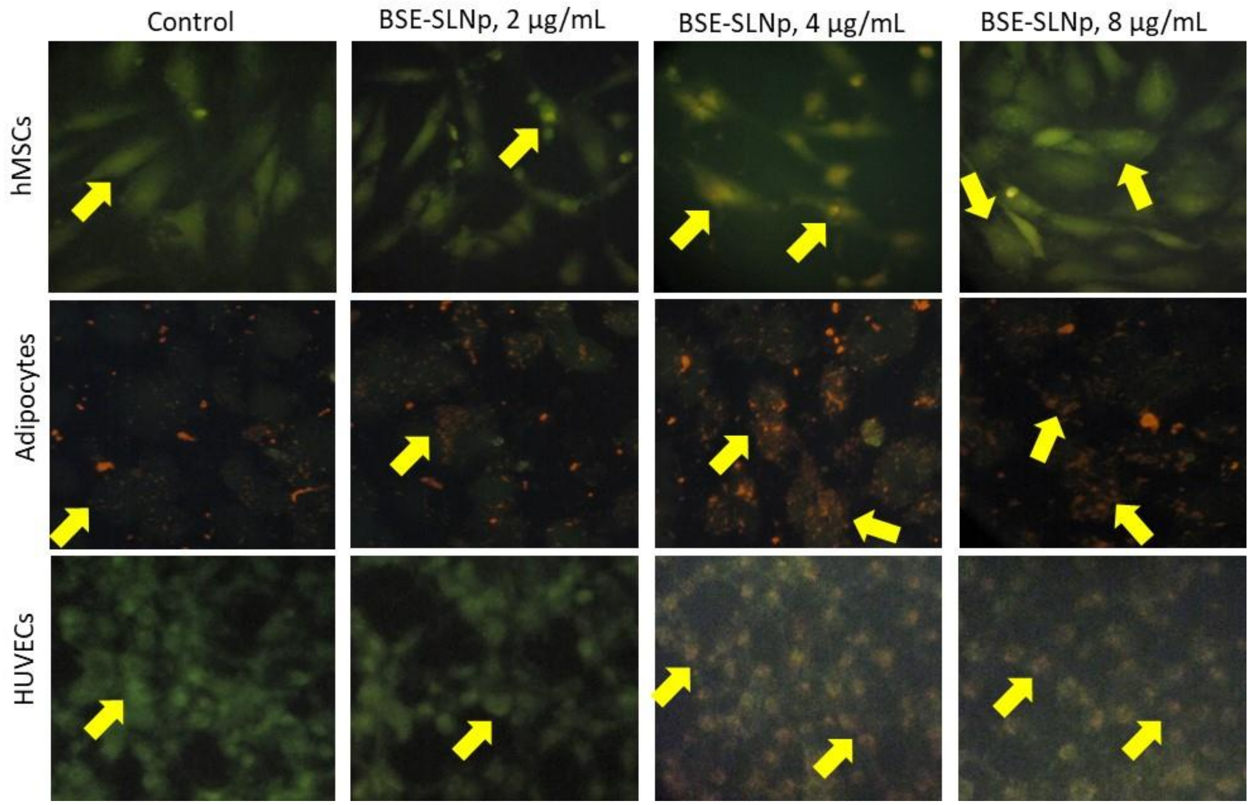

BSE-SLNp treated hMSCs, adipocytes and HUVECs showing the images of JC-1 staining clearly represent dye having red and green signal, comparable to J-aggregates vs. monomeric form. In vehicle control showing with less J-aggregates confirmed less mitochondrial potential than BSE-SLNp treated cells. Nevertheless, 4 μg/mL or 8 μg/mL of BSE-SLNp shown high J-aggregates compared to 2 μg/mL directly represent the highest capacity of BSE-SLNp on metabolic active cells with oxidative capacity and mitochondrial efficiency directly represent healthy cells. So, we have chosen 4 μg/mL as the effective dose with lowest concentration with highest activity.
